# Sensitivity and Specificity of CD19.CAR-T Cell Detection by Flow Cytometry and PCR

**DOI:** 10.3390/cells10113208

**Published:** 2021-11-17

**Authors:** Nicola Schanda, Tim Sauer, Alexander Kunz, Angela Hückelhoven-Krauss, Brigitte Neuber, Lei Wang, Mandy Hinkelbein, David Sedloev, Bailin He, Maria-Luisa Schubert, Carsten Müller-Tidow, Michael Schmitt, Anita Schmitt

**Affiliations:** Department of Internal Medicine V–Hematology, Oncology & Rheumatology, University Hospital Heidelberg, 69120 Heidelberg, Germany; nicola.schanda@t-online.de (N.S.); Tim.Sauer@med.uni-heidelberg.de (T.S.); Alexander.Kunz@med.uni-heidelberg.de (A.K.); Angela.Hueckelhoven-Krauss@med.uni-heidelberg.de (A.H.-K.); Brigitte.Neuber@med.uni-heidelberg.de (B.N.); Lei.Wang@med.uni-heidelberg.de (L.W.); Mandy.Hinkelbein@med.uni-heidelberg.de (M.H.); David.Sedloev@med.uni-heidelberg.de (D.S.); bailin.he@med.uni-heidelberg (B.H.); Maria-Luisa.Schubert@med.uni-heidelberg.de (M.-L.S.); Carsten.Mueller-Tidow@med.uni-heidelberg.de (C.M.-T.); Michael.Schmitt@med.uni-heidelberg.de (M.S.)

**Keywords:** CD19.CAR-T cells, flow cytometry (FACS), polymerase chain reaction (PCR), detection reagent

## Abstract

Chimeric-antigen-receptor-T (CAR-T) cells are currently revolutionizing the field of cancer immunotherapy. Therefore, there is an urgent need for CAR-T cell monitoring by clinicians to assess cell expansion and persistence in patients. CAR-T cell manufacturers and researchers need to evaluate transduction efficiency and vector copy number for quality control. Here, CAR expression was analyzed in peripheral blood samples from patients and healthy donors by flow cytometry with four commercially available detection reagents and on the gene level by quantitative polymerase chain reaction (qPCR). Flow cytometric analysis of CAR expression showed higher mean CAR expression values for CD19 CAR detection reagent and the F(ab’)2 antibody than Protein L and CD19 Protein. In addition, the CD19 CAR detection reagent showed a significantly lower median background staining of 0.02% (range 0.007–0.06%) when compared to the F(ab’)2 antibody, CD19 protein and Protein L with 0.80% (range 0.47–1.58%), 0.65% (range 0.25–1.35%) and 0.73% (range 0.44–1.23%). Furthermore, flow cytometry-based CAR-T cell frequencies by CD19 CAR detection reagent showed a good correlation with qPCR results. In conclusion, quality control of CAR-T cell products can be performed by FACS and qPCR. For the monitoring of CAR-T cell frequencies by FACS in patients, CAR detection reagents with a low background staining are preferable.

## 1. Introduction

Chimeric antigen receptor T (CAR-T) cell therapy constitutes an innovative and promising approach for the treatment of cancer that has fundamentally changed the field of immunotherapy [1]. The unparalleled 40% complete response rates in relapsed/refractory B-cell leukemia and lymphoma patients have resulted in the approval of commercially available CAR-T cell products, such as tisagenlecleucel (Kymriah™) and axicabtagene ciloleucel (Yescarta™) [2,3], and has resulted in the initiation of more than 100 clinical trials with CD19.CAR-T cells listed in the database ClinicalTrials.gov. In the context of CAR-T cell production and clinical application, accurate and reproducible CAR detection methods are required [4].

The basic structure of the chimeric antigen receptor comprises three main components: (1) the extracellular antigen-specific domain derived from an antibody’s single chain variable fragment (scFv); (2) the transmembrane domain; and (3) the intracellular domain that mediates downstream signaling. This basic structure remains as a standard, but with progress in CAR development, different generations of CARs have evolved. First-generation CARs contain only a single CD3ζ intracellular domain from the TCR/CD3 receptor complex. Second-generation CARs carry an added co-stimulatory domain, such as CD28 or 4-1BB, and third-generation CAR-T cells include two co-stimulatory molecules within their CAR constructs [5,6].

For CAR-T cell manufacturing in the commercial as well as in the academic setting, stringent regulations were implemented to ensure quality, safety and efficacy. The quality control assays have to be validated and require in vitro testing for the transduction efficiency of CAR-T cells and vector copy number per cell, which are according to current status performed by flow cytometry and by polymerase chain reaction (PCR).

In the clinical setting, monitoring of transfused CAR-T cells is crucial to determine persistence and proliferation, the two key mechanisms which define the anti-tumor efficacy of CAR-T cells in vivo [7,8]. Next to the impressive therapeutic potential, CAR-T cells can also induce significant toxicities [9]. Upon activation, CAR-T cells release cytokines, which can result in a potentially life-threatening cytokine release syndrome [10,11]. Additionally, tumor-targeting cells can also show an on target-off tumor effect, describing the attack of non-malignant tissue expressing the target antigen [12]. To clarify these side-effects, to elucidate non-response or relapse mechanisms and to develop CAR-T cell therapy, reliable detection methods are required. The most common methods used for the monitoring of infused CAR-T cells in a clinical setting are flow cytometry [13] and qPCR [14,15] using CAR-specific primers [16,17].

Quantitative PCR enables CAR vector detection on a genomic level, whereas flow cytometry allows to assess the protein expression of CAR molecules on the cell surface. Flow cytometry allows not only to assess the survival of CAR-T cells but also to perform a multi-parametric analysis for immunophenotypic characterization of these cells. With the expanding use of CD19.CAR-T cell products in the clinic, there is an urgent need for a reliable and cost-efficient detection method.

**Figure 1:**
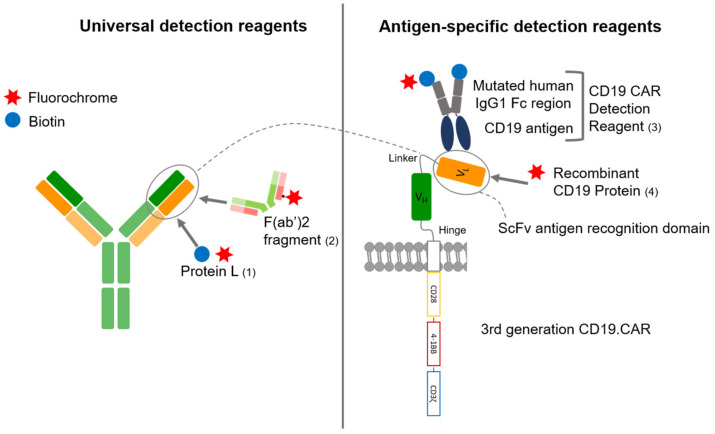
Binding mechanisms of the different CD19.CAR detection reagents. Universal detection reagents, Protein L (1) and F(ab’)2 fragment (2) are binding to the immunoglobulin light chain and to the Fab portion of the immunoglobulin. Antigen-specific detection reagents (3 and 4) are binding to the CD19 binding site of the scFv. The reagents are either directly conjugated to a fluorochrome as recombinant CD19 protein (4) and F(ab’)2 fragment (2) or are conjugated to biotin binding to an anti-biotin antibody or fluorochrome conjugated streptavidin in a second staining step as CD19.CAR detection reagent (3) and Protein L (1). The CD19.CAR consists of a CD3ζ cytoplasmatic domain fused to the CD28 and 4-1BB costimulatory domains. The light and the heavy chain variable domains (VL and VH) separated by a linker are building the single chain variable fragment (scFv), which is linked via a hinge-region to the transmembrane domain.

The aim of this study is to compare CAR-T cell frequencies measured by four different CAR-T cell detection reagents with flow cytometry, as well as to compare the two most commonly used techniques: flow cytometry and quantitative PCR. The flow cytometry detection reagents used in this work are either antigen specific or non-antigen specific, and either directly labeled with a fluorochrome or requiring a secondary staining. Information on binding mechanisms was collected from product data sheets unless stated otherwise. Figure 1 gives an overview on the different detection reagents used in this work.

Antigen-specific detection reagents bind to the antigen-binding domain of the CD19.CAR and are supposed to have a high specificity with a low background staining when compared to non-antigen specific detection reagents [18,19]. Yet, they are costly and can only be used for one particular scFv.

The CD19.CAR detection reagent (Miltenyi) constitutes a biotinylated antigen-based fusion protein which can be detected by an anti-biotin antibody conjugated to a fluorochrome. It consists of the human CD19 extracellular domains and a mutated human IgG1 Fc region. According to the manufacturer, the mutated human IgG1 Fc region does not require an Fc receptor blocking and allows staining with low background. The FITC-labeled human CD19 (20-291) protein (AcroBiosystems) is a recombinant protein. The target antigen is already pre-labeled with a fluorescent dye.

Universal CAR detection reagents, such as anti-Fab antibodies and Protein L, are non-antigen specific and can therefore be used for CARs of different design and antigen-specificity. Both target IgG-like fragments. While they are more cost effective, they might on the other hand be characterized by a higher background staining [19].

The anti-mouse IgG Fab antibody binds to the Fab portion of the class G immunoglobulins. By pepsin digestion of whole immunoglobulin, most of the Fc fragment has been removed, resulting in divalent F(ab’)2 fragments with two antigen-binding regions, linked by disulfide bonds. Due to the loss of the Fc fragment, non-specific binding to Fc receptors could be greatly reduced. As these are polyclonal antibody preparations, results might differ significantly between different batches.

Protein L, derived from Peptostreptococcus magnus, selectively binds to most subtypes of immunoglobulin light chains (κ chain) without interfering with the antigen-binding site [20]. The protein has a broad immunoglobin binding activity including an affinity for Fab fragments [21], regardless of the class-specific heavy chains. This has been shown by Zheng et al. for a variety of CARs, including anti-EGFRvIII, anti-CD19 and anti-HER2 [20]. It binds to single-chain antibody fragments (scFv) without interfering with the immunoglobulin’s antigen-binding site, enabling it to detect the cell surface expression of CARs [20,21]. It is important to include multiple washing steps to avoid carry-over of protein L in the washing buffer before staining with fluorochrome-conjugated streptavidin.

## 2. Materials and Methods

### 2.1. PBMCs from Healthy Donors and Patients

Buffy coats from healthy donors (HD) were obtained from the local blood bank (German Red Cross Blood Bank, Frankfurt am Main, Germany) after informed consent was given. Cells were isolated by density centrifugation using FicoLite-H (Linaris-H, Wertheim-Bettingen, Germany). Cell viability and cell number were determined by trypan blue staining (Sigma-Aldrich, St. Louis, MO, USA). Thereafter, PBMCs were frozen and cryopreserved in liquid nitrogen. Patient samples were acquired by leukapheresis after informed consent as part of the clinical HD-CAR-1 study from the University Hospital Heidelberg (EudraCT: 2016-004808-60) [22]. The patient CAR-T cells tested included samples from patients with r/r acute lymphoblastic leukemia (ALL) and r/r Non-Hodgkin’s Lymphoma. The CAR-T cell manufacturing was performed at the GMP Core Facility using a SFG.CAR.CD19.CD28.4-1BB.CD3zeta third-generation retroviral vector produced at the Baylor College in Houston, Texas (CAGT, Baylor College, Houston, TX, USA).

### 2.2. Manufacturing of 3rd Generation CD19.CAR-T Cells

CD19.CAR-T cells from HDs were manufactured by transducing PBMCs with a third-generation retroviral vector (SFG CD19.CD28.4-1BB.CD3zeta) supplied by the Baylor College in Houston, Texas (CAGT, Baylor College, Houston, TX, USA) followed by CAR-T cell expansion as previously described [23]. The production of the CD19-specific 3G (CD19.CAR.CD28.4-1BB.CD3zeta) retroviral CAR vector was performed by co-transfecting 293T cells with the specific retroviral vector plasmid, PegPam3 plasmid containing gag-pol and RDF plasmid containing the envelope followed by harvest of the generated retroviral supernatants. PBMCs were frozen down after Ficoll and thawed for the staining process. Primary donor cells were activated with anti-CD3/anti-CD28 antibodies (Biolegend, San Diego, CA, USA) and transduced with a CD19.CAR.CD28.4-1BB.CD3zeta retroviral vector. Cultivation was performed with IL-7/IL-15 (R&D Systems, Minneapolis, MN, USA). CD19.CAR-T cells were harvested on day 10 of expansion and directly stained or frozen down on day 14 of expansion. Patient samples were either stained on day 10 of expansion or frozen down. Staining was always performed on the same cell product of 1 donor for all detection reagents.

### 2.3. Flow Cytometry

Marker expression was evaluated by multicolor flow cytometry. The chimeric antigen receptor was stained using the following reagents: recombinant protein L was purchased from Thermo Fisher Scientific (Cat number 29997, Waltham, MA, USA) and reconstituted in ddH2O at 1 mg/mL; CD19 CAR detection reagent (biotinylated) was obtained from Miltenyi (Cat number 130-115-965) together with anti-biotin-PE (Cat number 130-090-756, Clone Bio3-18E7); FITC-labeled human CD19 (20-291) protein was purchased from AcroBiosystems (Cat number CD9-HF2H2, UniProtKB:P15391-1) and diluted with dH2O to a concentration of 100 μg/mL. Goat F(ab’)2 anti-human IgG (H+L)-RPE was bought from Jackson ImmunoResearch (Cat number 109-116-088, RRID:AB_2337676) and rehydrated in dH2O (1 mL). All reagents were stored at 4 °C. The following antibodies were used to stain for surface markers: CD3-BV510 (Biolegend, Clone OKT3), CD20-BV510 (Biolegend, Clone 2H7), CD14-APC (Biolegend, Clone 63D3), CD56-FITC (Biolegend, HCD56), CD45-PerCP (Biolegend, Clone 2D1), CD3-AF700 (Biolegend, Clone UCHT1), CD3-FITC (Biolegend, Clone UCHT1).

For FACS staining, 1 × 10^6^ cells were harvested and placed into a 5 mL round bottom polystyrene tube (Falcon™ 352054) and washed with cold PBS. Dead cells were excluded using the LIVE/DEAD Fixable Near-IR dead cell stain kit (Thermo Fisher Scientific). Cells were then washed with a flow buffer containing phosphate-buffered saline (PBS), pH 7.2, 0.5% bovine serum albumin (BSA) and 2 mM EDTA.

For Protein L (Thermo Fisher Scientific) staining, 1 μL of biotinylated Protein L was added in 0.1 mL of the wash buffer and incubated at 4 °C for 15 min. Cells were then washed with 2 mL of wash buffer and stained with 1 μL Streptavidin-Phycoerythrin (Sav-PE, 0.2 mg/mL) (Biolegend, Cat number 405203) in 0.1 mL of the wash buffer at 4 °C for 15 min in the dark.

For staining with the biotinylated CD19 CAR detection reagent (Miltenyi Biotec, San Diego, CA, USA), 2 μL were added in 0.1 mL of wash buffer and incubated at room temperature for 10 min in the dark. After washing with 2 mL of wash buffer, cells were stained with 1 μL anti-biotin-PE (Miltenyi, 130-090-756) in 0.1 mL of wash buffer and incubated at room temperature for 10 min in the dark.

For staining with FITC-labeled human CD19 (20-291) Protein (AcroBiosystems, Newark, DE, USA), 5 μL of antibody were added in 0.1 mL of wash buffer and incubated at 4 °C for 45 min in the dark. For staining with the goat F(ab’)2 anti-human IgG (H+L)-RPE (Jackson Immuno Research Labs, West Grove, PA, USA), 5 μL were added in 0.1 mL of wash buffer and incubated at room temperature for 30 min in the dark.

After staining with the respective antigen/antibody, the cells were washed again with the flow buffer, followed by data acquisition performed on a BD LSR II device (BD Biosciences, Franklin Lakes, NJ, USA). For data analysis FlowJo software (FlowJo™ Software for macOS, Version 10.8.0. Ashland, OR, USA: Becton, Dickinson and Company; 2021) was used.

To reliably define the CD19.CAR-T cell population, a minimum number of events is necessary. For evaluation of the different detection reagents in HDs and patient samples, a minimum number of at least 100,000 events was acquired. For assessment of sensitivity and specificity, the minimum number of events was 150,000.

### 2.4. Quantitative Real-Time PCR (qPCR)

qPCR results were acquired using a technology as previously published by our group [16]. Briefly, a single copy gene (SCG)-based duplex (DP)-qPCR assay (SCG-DP-PCR) was used to determine the vector copy number (VCN) in CAR-T cell products. To assess the VCN, 100 ng of genomic DNA was isolated from manufactured CAR-T cells and was amplified with StepOnePlusTM Real-time PCR system (Thermo Fisher Applied Biosystems, Waltham, MA, USA) using the respective primers, probes and TaqMan Gene Expression Master Mix. The following controls were included in all experiments: non-template control (NTC), biological negative control (non-transduced donor cells) and the RV-SFG.CD19.CD28.4-1BB.CD3zeta plasmid as positive control.

### 2.5. Data Analysis

No normalization was implemented in the analysis. All data were raw data as gated with FlowJo software and displayed in percentages CAR-T cells. Data were analyzed by standard statistical measures, arithmetic means and range (minimum/maximum). Statistical analysis was performed using Microsoft Excel^®^ and GraphPad Prism (GraphPad Prism version 9.2.0 for macOS, GraphPad Software, San Diego, CA, USA). Significance was determined via one-way ANOVA followed by a post-hoc Tukey multiple comparison test. In all tests, a *p*-value < 0.05 was considered to be statistically significant. Graphs were designed using Microsoft Excel^®^, Microsoft Word^®^ and GraphPad Prism.

## 3. Results

### 3.1. Comparison of Four Different Detection Reagents

Staining of manufactured CAR-T cells of five patients and five HDs was performed by four different CAR-T cell detection reagents. Samples were stained according to the staining protocol and a minimum of 100,000 events were acquired with a respective non-transduced control (Supplementary Material Figure S1). The positive CD19.CAR gate was set using a histogram setting, with the CAR positive and negative population separated by two different peaks.

**Figure 2:**
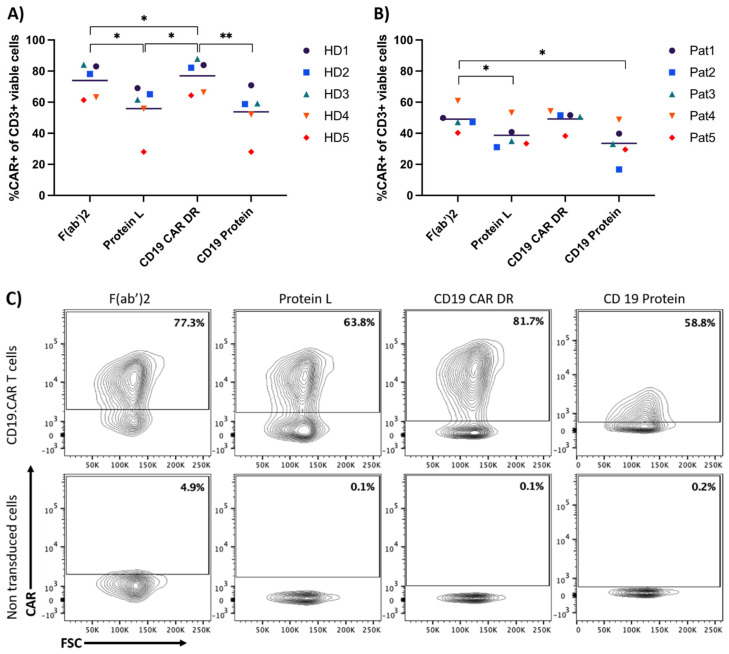
Comparison of four different antibodies to detect CD19-specific CAR-T cells. Both graphs show the different percentages for CAR-T cell detection when using the displayed staining reagents. (**A**) depicts the percentage of CAR-T cells produced from five different HD samples. (**B**) shows the percentage of CAR-T cells produced from five different patient samples (Pat). (**C**) shows the contour plots for CAR-T cells of one HD stained with all four different antibodies. The gate for the non-transduced cells is the same as in the respective CD19.CAR-T cell group. Comparison for HDs and patient samples were evaluated in three independent experiments respectively. (*) *p* < 0.05; (**) *p* < 0.01 by one-way ANOVA.

As shown in Figure 2, all reagents could detect the CAR with CD19 detection reagent, CD19 protein and protein L showing a clear discrimination between the positive and negative population. The percentage of CAR-T cells differed between the detection reagents. The CD19 CAR detection reagent and the F(ab’)2 fragment yielded the highest frequencies of CAR-T cells. These observations were consistent for CAR-T cells from healthy donors (HDs) as well as from patients.

### 3.2. Sensitivity

To examine the sensitivity and therefore the detection level of the different reagents, CD19-specific CAR-T cells were diluted in PBMCs of the respective donor at six different dilutions (1:0, 1:1, 1:5, 1:10, 1:50, 1:100, 1:1000). Experiments were repeated with CAR-T cells produced from four HDs. After the exclusion of dead cells, CD3+/CD14− cells were analyzed for CAR expression (Supplementary Material Figure S2). A minimum of 150,000 events were acquired by flow cytometry.

**Figure 3:**
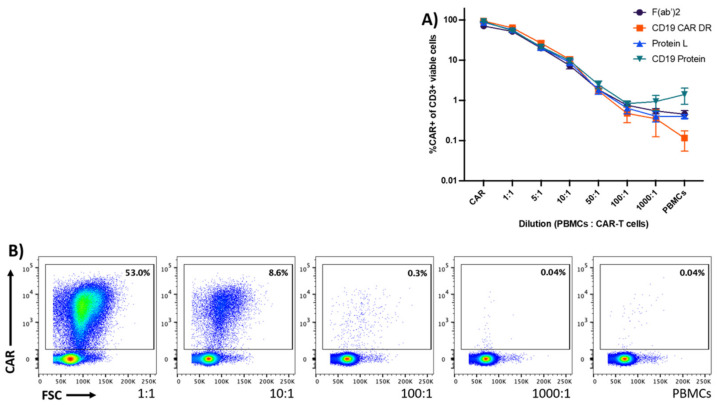
Sensitivity of the detection reagents to detect CD19-specific CAR-T cells in PBMCs. Graph (**A**) shows CD19-specific CAR-T cells that were serially diluted in PBMCs of the same HD at six different dilutions (1:1 to 1:1000). The graph displays the mean values ± standard error of mean of CAR-T cells to PBMCs from four HDs. (**B**) The dot plots display representative data obtained from one out of four different HDs. Data are representative of four different HDs acquired in one experiment.

All detection reagents showed a similar staining pattern throughout the dilutions 1:1 to 1:100 (Figure 3A). Both universal detection reagents and the CD19 protein displayed higher background staining in the 1:1000 and PBMC group. Even in very high dilutions (50:1, 100:1, 1000:1), the CD19 CAR detection reagent demonstrated a specific staining of CD19 CAR positive cells, as shown in Figure 3B.

### 3.3. Specificity

**Figure 4:**
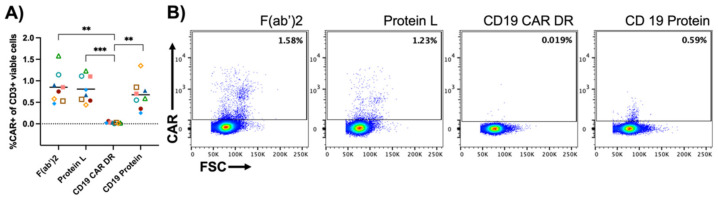
Specificity of the different detection reagents. PBMCs were stained with the respective CAR-detecting reagents to assess background staining. (**A**) shows the percentage of CD19.CAR-T cells in PBMCs only, for eight different donors. (**B**) displays results from one donor stained with different detection reagents. Data are representative of eight different HDs acquired in one experiment. (**) *p* < 0.01; (***) *p* < 0.001 by one-way ANOVA.

For the evaluation of unspecific binding, 1 × 10^6^ PBMCs from eight different donors were stained with the respective detection reagents and a minimum of 150,000 events were recorded. PBMCs were gated based on viability followed by the exclusion of CD14+, CD20+ and CD56+ cells. CD3+ cells were analyzed for CAR expression (Supplementary Material Figure S3). The F(ab’)2 antibody, CD19 protein and Protein L showed false-positive events when staining PBMCs only. In contrast, the CD19 CAR detection reagent showed almost no unspecific binding. The difference was highly significant (Figure 4).

### 3.4. Comparison of Flow Cytometry and qPCR for CD19.CAR-T Cell Detection

qPCR enables CAR detection on a genomic level and flow cytometry CAR expression on the cell surface. We compared the flow cytometry-based CAR-T quantification with a previously published qPCR-based method [16]. To that end, we generated CAR-T cells from three different *HDs* and performed a serial dilution (1:0, 1:1, 1:5, 1:10, 1:50, 1:100, 1:1000). The serial dilution was adjusted to the amount of CAR-transduced cells measured by the transduction efficiency.

FACS data were measured in % directly. Data from the qPCR experiments were transformed in % by dividing the copy number results of each dilution by the undiluted CAR T cell sample and multiplication with 100.

**Figure 5:**
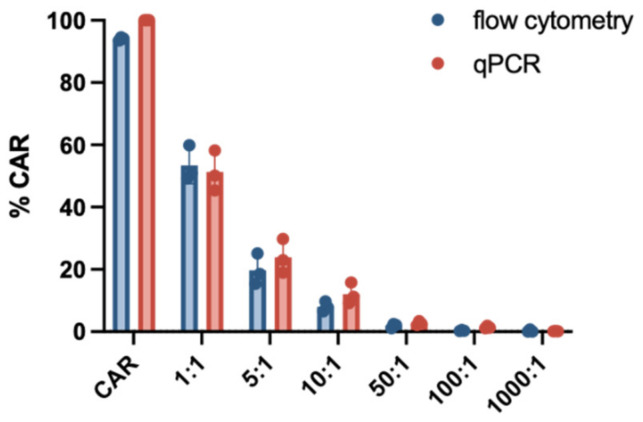
Comparison of CAR-T cell detection using flow cytometry and qPCR. CD19.CAR-T cells were diluted at six different dilutions (1:1 to 1:1000) and the percentage was compared with either flow cytometry using the CD19 CAR detection reagent or qPCR. Experiments were performed using three different HDs and yielded similar results. The graph displays the mean values and standard deviation.

As evidenced by the data shown in Figure 5, the results of both methods were concordant across the entire range of dilution. In most dilution groups, a slightly higher frequency of CAR T-cells could be measured when using qPCR, which could be due to background noise of non-viable cells. The measurement of transduction efficiency as outlined to this point was based on gating within the viable CD3+ population. However, our qPCR-based analysis uses the total amount of sample-derived DNA, including DNA from any nucleated cell component. For a more accurate comparison, this particular transduction efficiency was gated within the parent population of viable singlets as opposed to viable CD3+/CD14− cells.

## 4. Discussion

CAR T-cell therapy is considered a major scientific breakthrough in cancer immunotherapy. With the approval of adoptive CAR T-cell therapy, there is an urgent need for a reliable and time-saving method for the detection and analysis of CAR-T cells. This is of importance for clinicians for the monitoring of CAR-T cells in the peripheral blood of patients receiving CAR-T cell therapy, as well as for CAR-T cell manufacturing sites for quality control of CAR-T cell products. Furthermore, CAR-T cell analysis is also fundamental in the thriving field of pre-clinical research.

The ability to track CAR-T cells in the peripheral blood, as well as to analyze manufactured CAR-T cells, is crucial for the better understanding of clinical efficacy and to elucidate models for differences in clinical outcome and even therapy failure. Clinical efficacy is dependent on the expansion and persistence of CAR-T cells and therefore affects decisions with therapeutic relevance [19].

In this study, we analyzed CAR expression by different detection methods using flow cytometry and quantitative PCR. After the generation of CAR-T cells from HDs and patients’ peripheral blood samples, CAR-T cells were analyzed with four different detection reagents to optimize staining by flow cytometry.

For CAR-T cell detection antigen specific and non-antigen specific reagents were used. Commonly used non-antigen specific CAR staining reagents target IgG-like fragments, thus a broad range of different scFvs can be stained. Both Protein L and F(ab’)2 fragments are unexpensive staining methods. However, both methods show cross-reactivity with IgG-like proteins causing unspecific staining. Therefore, thorough washing and/or usage of FBS in the flow staining buffer is necessary. In addition, staining has to be conducted in several steps, avoiding contact between other T cell staining antibodies and the scFv staining [19].

Specific CAR-T cell detection reagents have been developed for the detection of transduced T cells that are engineered to express CARs on the cell surface, which recognize specific antigens. Due to the antigen specificity a low staining background is expected; however, the reagents are expensive and can be used for just one particular scFv.

Next to specificity and cost-effectiveness, staining time and versatility also play a role. Staining time is shortened by directly fluorochrome conjugated detection reagents, whereas biotinylated reagents need a second staining step with streptavidin conjugated to a fluorochrome or anti-biotin antibodies. This is more time-consuming, however more versatile concerning flow cytometry panel designs.

The aim of the present study was to compare different staining options using flow cytometry for CD19.CAR-T cells and to evaluate the conformity of cell percentage. CAR-T cells could be detected by all reagents and the overall protocol setup was easily manageable. Despite different staining techniques, all products allowed a time to result under two hours after sample reception.

Moreover, the CD19.CAR detection reagent showed a significantly lower background staining in PBMCs. This is especially relevant for patient samples with a rather low amount of CAR-T cells when compared to other nucleated cells; a problem that typically arises in samples of patients at later time points of follow-up after CAR-T cell therapy. Additionally, this detection reagent yielded the highest frequencies of CAR-T cells and allowed a reliable distinction of CAR-expressing cells and negative cells. This is of high importance in samples without a biological negative CAR control, which is often provided by a non-transduced sample in the academic setting but lacks for patient samples. Furthermore, the CD19-specific reagent allows the immunophenotypic distinction of different types of CARs and is easily compatible with antibody panels in multiparametric flow cytometry.

Next to flow cytometry, qPCR can be used for the analysis of CAR expression. qPCR can easily provide information on the genomic level regarding expansion and kinetics and also allows detection of very low amounts of CAR-T cells in the peripheral blood [14]. Maude et al. demonstrated detection of CD19-specific CAR-T cells in patients with sustained remission until 2 years post transfusion by using qPCR. Moreover, qPCR is required from the regulatory authorities to evaluate the safety profile of manufactured CAR-T cells by quantifying the so-called vector copy number (VCN)–the average vector copy per genome [3,24]. The combination of both techniques, flow cytometry and PCR, might be suggested for the distinction between a lack of CAR-T cell persistence and CAR downmodulation after antigen engagement [25,26]. Although qPCR is widely established in the clinical setting, the following disadvantages should be noted. With CAR expression being dependent on a variety of factors, such as DNA methylation, the chosen promoter and the promoter specificity, the amount of functional CAR-T cells is often overestimated with qPCR [19]. According to our data, the frequency of CAR-T cells stained by the CD19 detection reagent and measured by flow cytometry showed a good correlation with the qPCR results. Just a slightly higher frequency for CAR expression was seen for qPCR.

In conclusion, all CAR-detection reagents offered a reliable detection of CAR-T cells. For the quality control of CAR-T cell products by flow cytometry, CAR-T cell-detection reagents with a clear distinction between positive CAR expression and negative cells should be chosen. Additionally, for the monitoring of CAR-T cells in patient samples with rather low CAR-T cell frequencies, detection reagents with a low, unspecific binding should be favorized.

## Supplementary Materials

The following are available online at https://www.mdpi.com/article/10.3390/cells10113208/s1, Figure S1: Gating strategy for comparison of detection reagents. Representative gating strategy of a CAR-T cell sample from a HD for the analysis of different detection reagents. All viable, single cells were gated for CD3 positivity and then analyzed for CD19.CAR expression. Figure S2: Gating strategy for sensitivity measurement. Representative gating strategy of a CAR-T cell sample from a HD for the analysis of the sensitivity of different detection reagents. All viable, single cells were gated for CD3 positivity and CD14 negativity. This subpopulation was then gated for its CD19.CAR expression. Figure S3: Gating strategy for the assessment of specificity. Representative gating strategy of a peripheral blood sample from a HD for the analysis of the specificity of different detection reagents. All viable, single cells were gated for CD20 and CD14 negativity. These cells were then further analyzed for CD3 positivity and CD56 negativity and finally for its CD19.CAR expression.
