# Supplementary figures and images for: Sensitivity and Specificity of CD19.CAR-T Cell Detection by Flow Cytometry and PCR
Source: Cells. 2021 Nov 17;10(11):3208. doi: 10.3390/cells10113208 (PMC8621201; doi:10.3390/cells10113208)

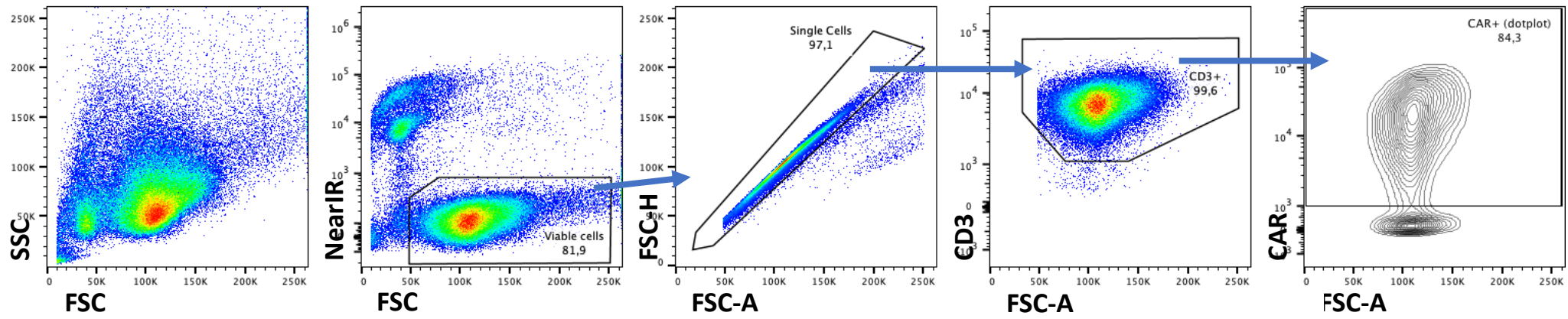

Supplement: Supplementary file 1 [file cells-10-03208-s001.zip › Supp New/Figure S1.pdf]

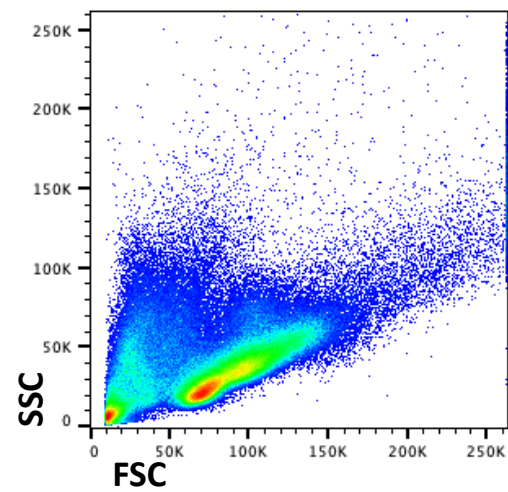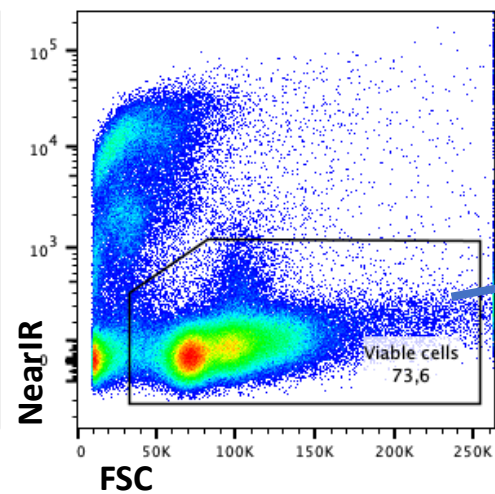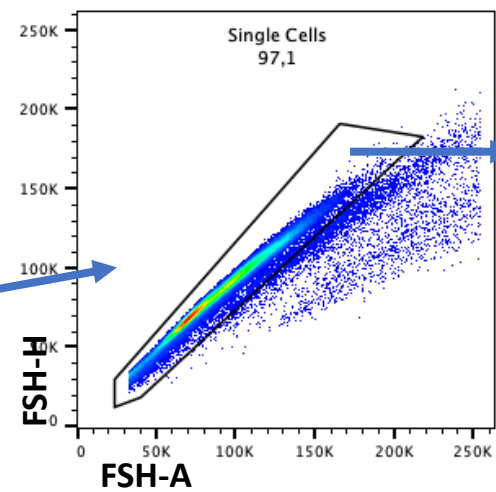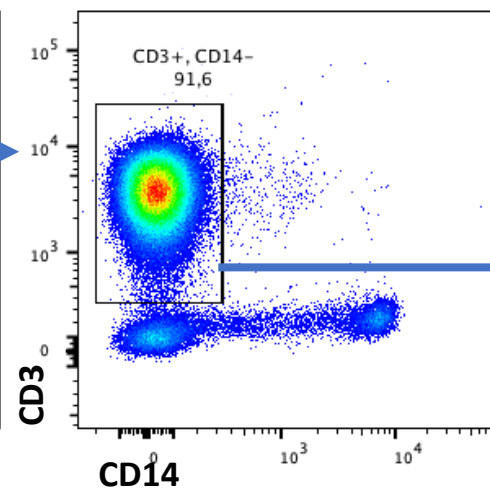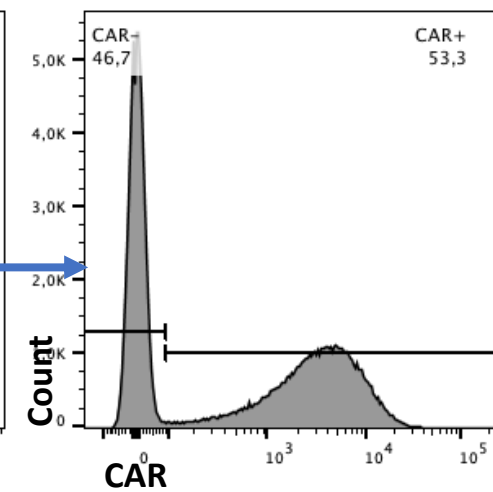

Supplement: Supplementary file 1 [file cells-10-03208-s001.zip › Supp New/Figure S2.pdf]

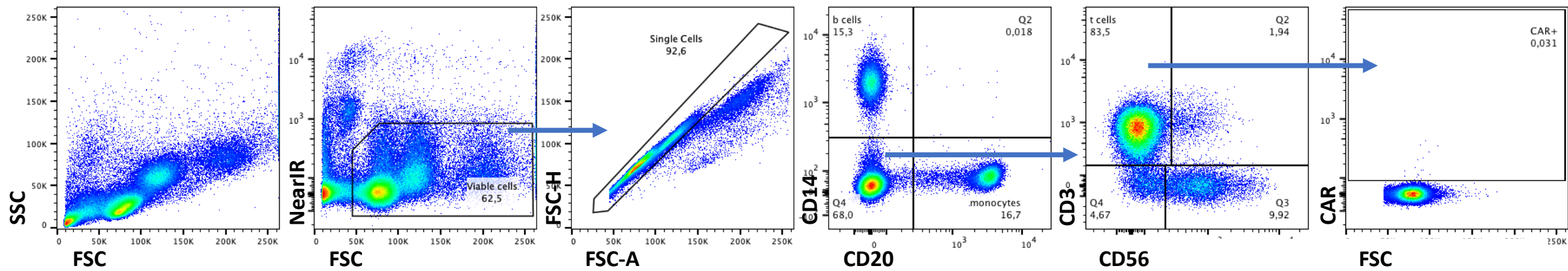

Supplement: Supplementary file 1 [file cells-10-03208-s001.zip › Supp New/Figure S3.pdf]
